# The mean and variance of climate change in the oceans: hidden evolutionary potential under stochastic environmental variability in marine sticklebacks

**DOI:** 10.1038/s41598-017-07140-9

**Published:** 2017-08-21

**Authors:** Lisa N. S. Shama

**Affiliations:** Alfred Wegener Institute Helmholtz Centre for Polar and Marine Research, Coastal Ecology Section, Wadden Sea Station Sylt, Hafenstrasse 43, 25992 List, Germany

## Abstract

Increasing climate variability may pose an even greater risk to species than climate warming because temperature fluctuations can amplify adverse impacts of directional warming on fitness-related traits. Here, the influence of directional warming and increasing climate variability on marine stickleback fish (*Gasterosteus aculeatus*) offspring size variation was investigated by simulating changes to the mean and variance of ocean temperatures predicted under climate change. Reproductive traits of mothers and offspring size reaction norms across four climate scenarios were examined to assess the roles of standing genetic variation, transgenerational and within-generation plasticity in adaptive potential. Mothers acclimated to directional warming produced smaller eggs than mothers in constant, ambient temperatures, whereas mothers in a predictably variable environment (weekly change between temperatures) produced a range of egg sizes, possibly reflecting a diversified bet hedging strategy. Offspring size post-hatch was mostly influenced by genotype by environment interactions and not transgenerational effects. Offspring size reaction norms also differed depending on the type of environmental predictability (predictably variable vs. stochastic), with offspring reaching the largest sizes in the stochastic environment. Release of cryptic genetic variation for offspring size in the stochastic environment suggests hidden evolutionary potential in this wild population to respond to changes in environmental predictability.

## Introduction

Ocean environments are warming at geologically unparalleled rates. Over the coming decades, average sea surface temperatures are predicted to increase by 2–5 °C^1^. The rate and scale of these directional changes in mean temperature have already resulted in alterations to marine species’ physiology and phenology, and to changes in the composition and distribution of communities^2^. More recently, the impact of increasing climate variability and changes to the frequency, duration and intensity of extreme climate events on species and population dynamics have gained attention^3, 4^. Both theoretical and empirical studies suggest that predictions based on directional changes to mean temperatures differ considerably from predictions that include changes to temperature variance, and that increasing variability of thermal environments might pose an even greater risk to population persistence and species extinction than climate warming^5–8^. The question now being asked is whether species can respond fast enough to keep pace with not only rapid warming, but also increasing climate variability^9^.

Persistence in the face of rapidly changing marine environments requires that populations harbour sufficient standing genetic variation (the raw material for evolutionary change)^10–13^ and/or phenotypic plasticity to mount a fast response^14–16^. Phenotypic plasticity can occur both within a generation (genotype by environment or G × E interaction) and across generations (transgenerational plasticity or TGP)^17^. TGP might be especially relevant under global change because it is a phenotypic response that can buffer populations against immediate impacts of changing environments and is inherited across generations, potentially buying time for genetic adaptation to catch up in the longer term^18^. Importantly, recent studies show that within-generation plasticity (WGP) can be altered by the environmental conditions experienced by previous generations (i.e. TGP)^19^, and that TGP can be modified or overridden by WGP^20^. In other words, the two forms of plasticity are not necessarily independent processes. Transgenerational plasticity may be selected for when environmental heterogeneity across generations is low, and parents can predict the environment their offspring will experience. Here, parents should produce offspring with a mean phenotype optimised for the predicted future environment^17, 21^. When future environmental conditions are unpredictable, and the potential for mismatch between parent and offspring environments is high, the evolution of bet hedging is expected. For example, parents may produce a range of offspring phenotypes with at least some having the optimal phenotype (diversified bet hedging) or larger than average offspring of presumably higher quality to withstand poor conditions (conservative bet hedging or playing it safe)^22–25^. Until recently, empirical evidence for bet hedging was scarce^24^; support for it as an adaptive strategy is starting to accumulate^23, 26–29^. Within-generation plasticity is also selected for in heterogeneous environments, but when environmental cues reflect the current state of the environment where selection acts on the phenotype^30^. From a within-generation perspective, environmental fluctuations may also generate optimal phenotypes and reaction norms that differ from those produced under constant conditions^31–33^ due to the nonlinear relationship between temperature and performance^34^, and to changes in the genetic and phenotypic variance available for selection to act on in constant versus fluctuating environments^35, 36^. Since evolutionary potential is influenced by all of the above (standing genetic variation, parental effects including TGP and bet hedging, as well as G × E interactions), an understanding of their interplay will be necessary to quantify the total adaptive potential of populations under directional climate warming and/or increasing climate variability.

In many taxa, size is a strong determinant of fitness and can respond rapidly to environmental change^37^. Theoretical models of optimal offspring size predict that a single optimal size will be favoured in constant environments, and if the size-fitness relationship changes across different constant environments, different sizes may be selected for in each environment^23^. Indeed, there are numerous examples of mothers shifting the phenotypes of their offspring via adaptive plasticity to match local conditions^38^. The size of individual offspring will also often trade-off with the number of offspring that can be produced^39^. When environmental conditions are good, some models predict that mothers should maximise fecundity by producing many, small offspring, whereas in adverse environmental conditions they should produce fewer, large offspring of presumably higher quality^22^. However, bigger is not always better in bad environments^40^, for example, if large size is associated with higher physiological demands in stressful conditions^26^. In unpredictable environments, models predict that mothers could hedge their bets for offspring size via two different strategies: diversified bet hedging^25^ or conservative bet hedging^24^. Additionally, the type of environmental predictability can also influence offspring size. Periodic fluctuations, like seasonality or tidal cycles, with regularity in the timing and magnitude of changes around the average environmental state may have very different effects on offspring size than environmental noise (or environmental colour), whereby predictability is determined by the degree to which the environment is similar between successive time points (autocorrelation)^41^. In predictably varying environments, predictability is about the mean environmental state, whereas in noisy or stochastically varying environments, predictability is about how long an environmental state persists^41^. Furthermore, Jensen’s inequality for nonlinear functions predicts that organism responses to fluctuating environments will not be symmetrical, with stronger effects due to increased compared with decreased temperatures^42^, although the opposite pattern has also been found^33^. Therefore, how long the environmental state persists above versus below the seasonal mean will also play a role in determining offspring size^43^. Few empirical studies to date have simultaneously investigated different types of predictability on offspring size variation (but see refs 33, 36 and 44); hence, current offspring size theory is lacking an integrative understanding of the influence of environmental predictability^41^.

The main goal of this study was to assess the potential for offspring size variation in both directionally warming and fluctuating environments by simulating changes to the mean and variance of sea surface temperatures (SST) predicted under climate change^1^ using the marine threespine stickleback fish (*Gasterosteus aculeatus*) as a model. The threespine stickleback (herein referred to simply as stickleback) is an ideal model for investigating evolutionary processes in general, and behavioural, morphological, and life history plasticity specifically. Stickleback occur in marine, brackish and freshwater environments throughout the northern hemisphere and can be easily bred and reared under laboratory conditions. Their extensive intraspecific variation coupled with a comprehensive genomic toolbox make them highly amenable for studies of the adaptive value of particular phenotypes^39^. Here, the role of maternal environment effects on reproductive traits and offspring size was investigated by acclimating stickleback mothers to either constant, predictably variable or stochastically varying temperatures to determine whether they can adjust how resources are allocated to offspring, and if within-clutch variation in offspring size changes in response to changes in mean temperature and temperature variability. Similarly, the roles of TGP and WGP (G × E interactions) were investigated by rearing sibling offspring in each experimental climate scenario to determine the shape of their (family) thermal reaction norms and to test the extent of genetic variation for plasticity of offspring size. The population studied here inhabits an area of the North Sea with a mean SST of 17 °C during summer months, but with abrupt changes in temperature direction occurring at irregular intervals^29^. Previous studies found that exposure to a constant elevated temperature of 21 °C (simulated in accordance with a 2100 climate scenario^45^) reflected a chronic stress for this population that had negative effects on growth, development and survival compared to ambient (17 °C) conditions^46–49^. When reared at 21 °C, offspring had lower hatching success^48^, reached smaller sizes^49^, had higher shape variance^46^ and higher mortality when exposed to pathogens than when reared at 17 °C^47^. Yet, when mothers were acclimated to constant 21 °C during reproductive conditioning (two months just prior to spawning), TGP in response to predictable environmental cues of future warming resulted in (relatively) larger offspring at 21 °C^48^. However, when stickleback were acclimated to unpredictable environments where the mean temperature switched weekly between 17 °C and 21 °C, mothers produced more variably sized offspring, suggesting that they may have used a diversifying bet-hedging strategy to cope with the variable environment^29^. But how mothers respond to stochastically varying environments may differ from their response in predictably varying conditions. For instance, SST can change by several degrees very quickly depending on erratic weather patterns and less frequent but more extreme events such as heat waves or cold snaps^4, 6^. Here again, mothers should hedge their bets, but whether they should employ diversifying or conservative bet hedging has not been investigated. Moreover, predictions for optimal offspring size in stochastic environments are equivocal (increase, decrease or no change), and few direct tests of the role of environmental predictability in driving offspring size variation have been conducted to date^41^.

Here, the prediction was that the mean and variance of offspring phenotypes should differ depending on the mean, variance and predictability of the thermal environment. Specifically, mothers acclimated in constant environments should produce offspring of a mean optimal size to match local thermal conditions (as in ref. 49), and TGP in offspring size should be favoured since mothers can predict their offspring’s thermal environment. In fluctuating environments that change with predictable variability (weekly change between two temperatures), mothers should produce a range of offspring sizes (as in ref. 29) that include the optimal size for both temperatures, leading to a higher variance in offspring size and a mean size that is intermediate between the two. For mothers acclimated to stochastically varying temperature environments, the variance among offspring phenotypes is also predicted to be large, but no *a priori* predictions for mean offspring size are made. Finally, as environmental predictability will interact with WGP (G × E interactions) in complex ways^31^ due to nonlinear temperature performance curves and Jensen’s inequality^43^, the extent to which offspring rearing environment modifies maternal influences on offspring size is also likely to differ among thermal environments.

## Results

### Egg size was significantly influenced by maternal environment

**Figure 1:**
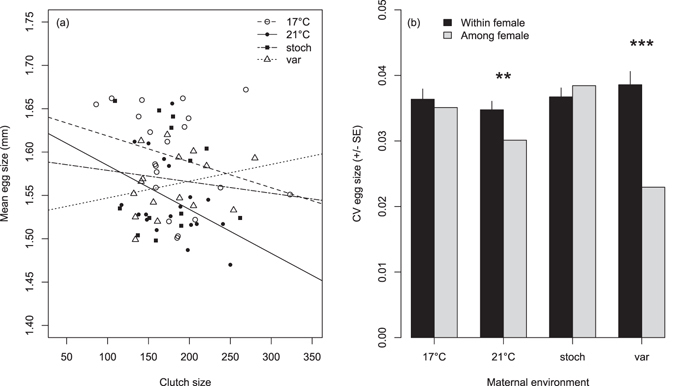
Mean egg size, clutch size, and egg size variability of female stickleback (*Gasterosteus aculeatus*) acclimated to the four experimental temperature treatments (constant 17 °C, constant 21 °C, predictably variable, and stochastically variable). (**a**) Relationship between clutch size (total number of eggs per female) and mean egg size (diameter ± 0.01 mm of 40 eggs per female) in the four treatments (Note: lines connect clutches within each maternal environment), and (**b**) egg size variability estimated by the coefficient of variation (CV) of egg size depicted as within-female variability (black bars) and among-female variability (grey bars), **P < 0.01, ***P < 0.001.

Maternal acclimation environment, female size, and clutch size significantly influenced mean egg size (Table 1). Mothers acclimated to 21 °C produced smaller eggs (mean egg diameter (mm) ± sd: 1.545 ± 0.070) than mothers acclimated to 17 °C (1.593 ± 0.080), stochastic (1.570 ± 0.081), and predictably variable environments (1.563 ± 0.068; Fig. 1a). To determine if egg size differences among treatments were driven by maternal acclimation environment and not female size, a second generalised linear mixed model (GLMM) was run using egg size residuals (egg size corrected for female size) as the response variable, which showed that significant factors in the full model remained significant when accounting for female size (significant clutch size: F_1,64_ = 7.21; *P* = 0.009 and maternal environment effects: Dam °C F_3,64_ = 3.44; *P* = 0.022). Importantly, female size also did not differ significantly among acclimation treatments (F_3,64_ = 0.66; *P* = 0.581). In a full GLMM, clutch size was significantly influenced by female size and egg size, but not by maternal environment (Table 1). Residual clutch size (clutch size corrected for female size) showed the same pattern, with a significant effect of egg size (F_1,64_ = 6.90; *P* = 0.011), but not maternal environment (F_3,64_ = 0.16; *P* = 0.924). Overall, there was a negative relationship between mean egg size and clutch size (Fig. 1a), but no significant difference among maternal environment slopes (clutch size/egg size × Dam °C p > 0.05; Table 1).

**Table 1:** Mean egg size, clutch size and the coefficient of variation (CV) of egg size for stickleback (*Gasterosteus aculeatus*) mothers acclimated to the four temperature treatments (Dam °C) analysed using generalised linear mixed effects models (GLMMs). Female (individual) was modelled as a random effect, Dam °C was modelled as a fixed effect, and female size, clutch size or egg size were included as covariates.

	Mean egg size			Clutch size			CV of egg size		
	denDF	*F*	*P*	denDF	*F*	*P*	denDF	*F*	*P*
(Intercept)	2581	76568.0	<0.001	60	1470.07	<0.001	59	2334.58	<0.001
Female size	60	6.31	0.014	60	16.77	<0.001	59	0.31	0.574
Clutch size	60	8.39	0.005				59	0.62	0.424
Egg size				60	7.38	0.009			
Dam °C	60	2.83	0.045	60	0.16	0.922	59	1.55	0.195
Clutch size × Dam °C	60	0.91	0.440				59	0.17	0.915
Egg size × Dam °C				60	0.94	0.425			

Numerator degrees of freedom were 1 in all cases except for Dam °C and the interaction terms (numDF = 3). denDF indicates denominator degrees of freedom. Note: no model selection was performed, hence, results reflect a full model including all fixed and random effect terms.

Within-female egg size variability (CV of egg size) was not significantly influenced by female size, clutch size, or maternal environment (Table 1). Within-female CVs of egg size were highest in the predictably variable environment (mean 0.0385 vs. 0.0365 in stochastic, 0.0362 at 17 °C, and 0.0338 at 21 °C), but error distributions overlapped with other treatments (Fig. 1b). A *post hoc* power analysis using the ‘pwr’ package in R showed that Cohen’s *d* effect sizes were 0.398, 0.048 and 0.314 (calculated using the mean and standard deviation of egg size CV for pairwise contrasts treating 17 °C as the control e.g. 17 °C–21 °C; 17 °C – stochastic; 17 °C – predictably variable, respectively). Effect sizes of this magnitude (0.3) are considered moderate^50^. Similarly, estimating effect size as *η* ^2^ using all group means (variance explained in an ANOVA) revealed a medium effect size of 0.074. Based on a Cohen’s *d* effect size of 0.3 and an ANOVA approach power estimation, a sample size of n = 32 females per acclimation environment would have been needed to reach a power level above 0.8^50^. Here, the number of females in each environment ranged between n = 15 and n = 19. In general, however, females in the predictably variable environment produced a broader range of egg sizes within a clutch than females in the constant and stochastic environments, which tended to produce clutches composed of either small or large eggs (Supplementary Fig. S1). Also, among-female CV of egg size was lowest for predictably variable environment mothers, followed by 21 °C, 17 °C and stochastic environment mothers (Fig. 1b). That is, individual females in the predictably variable environment produced clutches with a broader range of egg sizes and more similar clutches to one another than females in the other treatments (Supplementary Fig. S2), leading to the highest within-female and lowest among female variation of egg size in the predictably variable environment. Specifically, within-female egg size variability differed significantly from among-female variability in the predictably variable (*t* = 7.69; *P* < 0.001) and 21 °C environments (*t*-test: *t* = 2.93; *P* = 0.009), but not in the 17 °C (*t* = 0.71; *P* = 0.487) and stochastic environments (*t* = 1.49; *P* = 0.156; Fig. 1b).

### Genotype by environment interactions had the strongest effects on offspring body size

**Figure 2:**
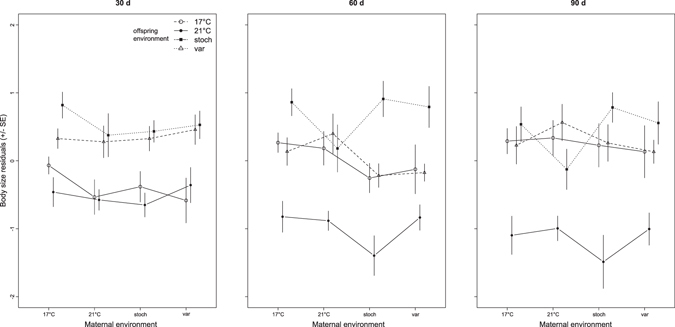
Body size residuals (standard length (mm) corrected for density) of stickleback (*Gasterosteus aculeatus*) offspring at 30, 60 and 90d post-hatch in the four maternal acclimation and offspring rearing temperature environments (constant 17 °C; open circles, constant 21 C; closed circles, predictably variable; open triangles, and stochastically variable; closed squares). Points reflect means (±SE) of all families within each maternal-offspring temperature combination; lines join offspring rearing environments.

**Figure 3:**
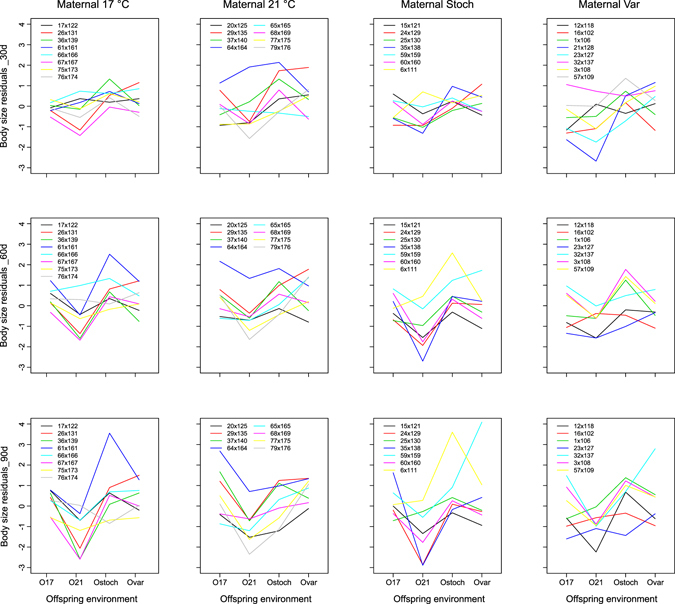
Family reaction norms of body size residuals (standard length (mm) corrected for density) of stickleback (*Gasterosteus aculeatus*) offspring at 30, 60, and 90 d post-hatch in the four maternal acclimation environments (four panels) and offspring rearing temperature environments (constant 17 °C, constant 21 C, predictably variable, and stochastically variable). Family names are shown as sire × dam identification numbers; lines join offspring rearing temperatures.

Density (number of offspring individuals in an aquarium) had a significant effect on offspring body size at all three time points (Table 2). Densities ranged between 5 and 12, and had a clear effect on offspring growth (smaller size at higher density), but the range of densities in the different offspring environments overlapped (Supplementary Fig. S3). Also, density × offspring °C interactions were not significant at 30d (F_3,157_ = 2.66; p = 0.051), 60d (F_3,156_ = 1.13; p = 0.339), or 90d (F_3,156_ = 0.46; p = 0.711), indicating that any potential effects of density on offspring body size were similar in all treatments. Note: At 30d, body sizes in the 17 °C offspring environment were less affected by density than in the other temperature treatments, driving the nearly significant density × offspring °C interaction (Supplementary Fig. S3). By far, offspring rearing environment had the clearest influence on stickleback body size (Table 2). At all three time points, offspring were larger when reared in the stochastic environment and smaller when reared at 21 °C, whereas body sizes were similar in the 17 °C and predictably variable environments (Fig. 2). At 30d, mean standard lengths (mm ± sd) for each offspring environment (averaged across maternal environments) were: 15.632 ± 1.082, 15.353 ± 1.206, 16.237 ± 1.329, and 16.159 ± 1.121 in the 17 °C, 21 °C, stochastic and predictably variable environments, respectively. At 60d, mean standard lengths were 19.486 ± 1.395, 18.371 ± 1.521, 19.776 ± 1.814, and 19.252 ± 1.665 in the 17 °C, 21 °C, stochastic and predictably variable environments, respectively. At 90d, mean standard lengths were 21.527 ± 1.874, 20.152 ± 1.890, 21.366 ± 2.158, and 21.297 ± 2.032 in the 17 °C, 21 °C, stochastic and predictably variable environments, respectively. Maternal acclimation environment (Dam °C) did not have a significant overall effect on offspring size, nor did the interaction between maternal and offspring temperatures (TGP). Rather, G × E interactions (family × offspring °C) strongly influenced offspring size at all three time points (Table 2, Fig. 3). Log-likelihoods of models with and without the family × offspring interaction term, respectively, were −1750.9 vs. −1808.1 at 30d, −1961.5 vs. −1987.8 at 60d, and −2091.4 vs. −2130.8 at 90d post-hatch, reflecting highly significant effects of G × E interactions on offspring size.

**Table 2:** Stickleback (*Gasterosteus aculeatus*) offspring body size (standard length) at 30, 60 and 90 d post-hatch analysed using GLMMs depicting the influence of density, egg size, maternal acclimation temperature (Dam °C), offspring rearing temperature (Offspring °C), and their interaction (Dam °C × Offspring °C). Chi square test statistics are given (Chisq.), along with degrees of freedom (Df) and associated p-values (Pr(>Chisq)). The random effect interaction term family (nested within Dam °C) by offspring temperature (Family × Offspring °C) and its Chi square statistics based on likelihood ratio tests between the full and reduced model are also shown.

	Size 30 days			Size 60 days			Size 90 days		
	Chisq.	Df	Pr(>Chisq)	Chisq.	Df	Pr(>Chisq)	Chisq.	Df	Pr(>Chisq)
*Fixed effects*									
Density	185.778	1	<0.001	450.074	1	<0.001	829.811	1	<0.001
Egg size	0.766	1	0.381	1.551	1	0.213	1.631	1	0.207
Dam °C	0.418	3	0.937	3.763	3	2.288	0.998	3	0.802
Offspring °C	82.178	3	<0.001	141.361	3	<0.001	96.502	3	<<0.001
Dam °C × Offspring °C	4.607	9	0.867	12.864	9	0.169	13.080	9	0.159
*Random effects*									
Family × Offspring °C	114.520	10	<0.001	52.768	10	<0.001	78.681	10	<0.001

Note: no model selection was performed, hence results denote a full model including all fixed and random effect terms.

Multivariate animal models for offspring size at 30, 60 and 90d fit best when family was included as a random effect, and density, egg size, Dam °C and offspring °C (but not the Dam °C × offspring °C term) were included as fixed effects, confirming the results found for the full data set (Supplementary Table S1). A large and significant proportion of the variation in offspring size was attributable to genetic (family) effects with ΔDICs of −239.4 at 30d, −206.0 at 60d, and −238.9 at 90d when family was included in the models. At all three time points, variance components differed among offspring rearing environments (Table 3). At 30d post-hatch, genetic variance (V_G_) and total phenotypic variance (V_G_ + V_E_ = V_P_) among families were highest at 21 °C. At 30d, genetic variance was 41.6% higher at 21 °C than in the next-highest treatment (predictably variable). At 60d post-hatch, V_G_ and V_P_ were highest in the stochastic environment, with V_G_ 35.1% higher in the stochastic treatment than in the next-highest treatment (21 °C). At 90d, V_G_ and V_P_ were again highest in the stochastic environment. Genetic variance in the stochastic treatment was 32.7% higher than in the next-highest treatment (predictably variable; Table 3). Environmental variance (V_E_) was similar among offspring temperatures at each time point, but decreased over time (Table 3). Non-significant genetic correlations between offspring environments (indicating G × E) were detected for almost all environment combinations at some time point. Only the correlation between 17 °C and stochastic offspring environments was significant at 30, 60 and 90d post-hatch (Table 3).

**Table 3:** Genetic variance-covariance matrices for stickleback (*Gasterosteus aculeatus*) offspring body size (standard length) at 30, 60 and 90 d post-hatch analysed by multivariate animal models taking temperature-specific size (character states) at 17°C, 21 °C, stochastic (Stoch), and variable (Var) offspring rearing environments as response variables.

	17 °C	21 °C	Stoch	Var
Size 30 days				
17 °C	V_G_ 0.272 (0.114–0.464)	*CoV 0*.*143* (*0*.*029*–*0*.*382*)	*CoV 0*.*047* (*−0*.*018*–*0*.*236*)	*CoV 0*.*042* (-*0*.*096*–*0*.*207*)
	V_E_ 0.416 (0.348–0.509)	*rG 0*.540* (*0*.*194*–*0*.*817*)	*rG 0*.468* (*0*.*013*–*0*.*739*)	*rG 0*.*106* ^*ns*^ (−*0*.*305*–*0*.*565*)
21 °C		V_G_ 0.405 (0.201–0.746)	*CoV 0*.*077* (*−0*.*033*–*0*.*290*)	*CoV 0*.*008* (*−0*.*203*–*0*.*164*)
		V_E_ 0.494 (0.412–0.613)	*rG 0*.*444* ^*ns*^ (*−0*.*040*–*0*.*707*)	*rG 0*.*026 * ^*ns*^ (*−0*.*465*–*0*.*369*)
Stoch			V_G_ 0.192 (0.101–0.393)	*CoV 0*.*065* (*−0*–*060*–*0*.*216*)
			V_E_ 0.503 (0.414–0.607)	*rG 0*.*261 * ^*ns*^ (*−0*.*155*–*0*.*660*)
Var				V_G_ 0.286 (0.150–0.570)
				V_E_ 0.399 (0.332–0.490)
Size 60 days				
17 °C	V_G_ 0.168 (0.081–0.364)	CoV 0.080 (−0.003–0.207)	CoV 0.115 (0.017–0.266)	CoV 0.087 (0.004–0.230)
	V_E_ 0.302 (0.255–0.376)	rG 0.372* (0.026–0.723)	rG 0.623* (0.205–0.822)	rG 0.569* (0.150–0.792)
21 °C		V_G_ 0.185 (0.092–0.374)	CoV 0.103 (−0.008–0.243)	CoV 0.040 (−0.035–0.190)
		V_E_ 0.345 (0.289–0.435)	rG 0.479* (0.068–0.747)	rG 0.294 ^ns^ (−0.113–0.701)
Stoch			V_G_ 0.250 (0.130–0.496)	CoV 0.087 (−0.012–0.244)
			V_E_ 0.332 (0.265–0.388)	rG 0.548* (0.081–0.786)
Var				V_G_ 0.178 (0.083–0.362)
				V_E_ 0.327 (0.270–0.398)
Size 90 days				
17 °C	V_G_ 0.172 (0.091–0.341)	CoV 0.019 (−0.074–0.109)	CoV 0.058 (−0.020–0.199)	CoV 0.084 (0.023–0.228)
	V_E_ 0.224 (0.182–0.267)	rG 0.115 ^ns^ (−0.329–0.507)	rG 0.399* (−0.023–0.676)	rG 0.576* (0.223–0.802)
21 °C		V_G_ 0.162 (0.075–0.304)	CoV 0.047 (−0.036–0.185)	CoV 0.032 (−0.050–0.132)
		V_E_ 0.246 (0.203–0.313)	rG 0.319 ^ns^ (−0.094–0.698)	rG 0.191 ^ns^ (−0.212–0.609)
Stoch			V_G_ 0.239 (0.122–0.444)	CoV 0.090 (0.015–0.236)
			V_E_ 0.226 (0.184–0.270)	rG 0.540* (0.157–0.764)
Var				V_G_ 0.180 (0.089–0.346)
				V_E_ 0.241 (0.200–0.298)

Elements on the diagonal give estimated genetic (V_G_) and environmental (V_E_) variance components with 95% CI. Off-diagonal elements give genetic covariances (CoV) and genetic correlations (rG) between character states. Significance of genetic correlations was tested as the proportion of posterior values overlapping zero. *P < 0.05; ns = not significant.

## Discussion

The most striking findings of this study are that environmental predictability plays a major role in marine stickleback offspring size variation, and that mothers can allocate resources to eggs differently depending on the mean and variance of the thermal environment they experience just prior to egg laying. In addition, offspring size reaction norms strongly depended on the type of environmental predictability (predictably variable vs. stochastic) they experienced. Finally, release of cryptic genetic variation for offspring size when reared in stochastically fluctuating environments suggests hidden evolutionary potential of this population to respond to the predicted increase in ocean climate variability.

### Maternal thermal environment mean and variance shape egg size allocation

Stickleback mothers adjusted the allocation of resources to eggs depending on the environment they experienced just prior to spawning, during the last phases of reproductive conditioning and egg maturation. On average, females acclimated to a constant mean temperature of 21 °C produced smaller eggs than females acclimated to a constant mean of 17 °C, and to both predictably variable and stochastic temperature regimes. Mean egg size also traded-off with clutch size, in that larger clutches were comprised of smaller eggs, and this was most evident for females acclimated to 21 °C (Fig. 1a). Female size is considered to be the best predictor of reproductive output in stickleback^39^. Here, egg allocation varied with maternal acclimation environment independent of female size, suggesting that the egg size plasticity shown was likely not due to physical size constraints^51^, but possibly reflects selection for different sized offspring in different environments^23^. Egg size plasticity in response to oviposition temperature has been demonstrated in numerous taxa^52, 53^, and larger eggs from females acclimated to 17 °C versus smaller eggs from females acclimated to 21 °C has been shown in two previous studies of this population^29, 49^. Large offspring from mothers acclimated to cold conditions is a common pattern of egg size variation, and may be selected for if larger offspring perform better at lower temperature^52^. Likewise, there is evidence suggesting that smaller eggs may be advantageous in warm conditions due to their lower oxygen demands^40, 54^. In both cases, such egg size plasticity would constitute adaptive TGP^17, 55, 56^. However, this will depend on relative embryo and yolk sizes, in conjunction with their specific oxygen demands and their relationship to offspring performance in different environments^57^, which remains to be tested for stickleback. Alternatively, females acclimated to a stressful constant 21 °C environment produced smaller eggs in favour of fecundity (selfish maternal effect *sensu* ^55^), enabling them to maximise their own fitness over offspring fitness^58^. Variation in egg size and clutch size are discussed here in terms of maternal thermal environment (see also ref. 59), but how eggs are matured/allocated just prior to spawning can depend on a number of factors such as photoperiod^60^, salinity ^61^, food availability (i.e. condition of the female^39^), age of the female^62^, predators^63, 64^ and parasites^65^. Within the female, physiological processes like stress hormone^63^, yolk steroid^66^, testosterone^67^ and carotenoid production^68^, as well as trade-offs with other traits like lateral plate production^60^ and immune response^69^ can also lead to variation in features of eggs and clutches. For the marine stickleback population studied here, little is known about habitat variability in the open sea where they overwinter and where the early stages of ova maturation begin^60^, but adults aggregate into small tidal channels and salt marsh pools for spawning starting in late spring (just prior to the last phases of egg maturation) where the habitat conditions (biotic and abiotic) are similar for all females (Shama, pers. obs.).

Females in the predictably variable environment produced a broad range of egg sizes within a clutch that encompassed the optimal egg size at both 17 °C and 21 °C, whereas females in the constant and stochastic environments tended to produce clutches composed of either small or large eggs. There was variation among females within each acclimation environment, but females in the predictably variable environment tended to respond to their environment in the same way (lowest among-female variation), whereas females in the other treatments did not (Fig. 1b). In unpredictable environments, within-female variability is expected to be higher than among-female variability, and such a pattern may indicate a diversified bet hedging strategy by females^23, 26^. The variability in egg size shown here fits well to this for the predictably variable environment but not the stochastic environment. In the predictably variable environment, mothers experienced the same temperature for a week, which is within the predictability range (6 to 9 days) experienced in the wild^29^. Since the time necessary for the final stages of egg maturation likely spanned more than one week^60^, females in this environment may have hedged their bets by producing a range of egg sizes, thereby spreading the risk of incorrectly predicting future environmental conditions^25^. The temperature conditions that hatched fry actually experience, however, are likely to be more complex. For instance, hatchlings from a mother exposed to elevated temperatures just prior to egg laying will experience ambient temperatures, but only for a day or two (depending on the time to hatching) during early development when yolk reserves are still present. After these initial days, the environment in which fry begin to feed exogenously will have switched back to match the maternal environment. The stochastic environment, however, was likely too unpredictable, in that the duration of any one temperature was rarely longer than a few days, and females essentially had no reliable environmental cue on which to base their egg allocation^33, 41^. As mean egg size was second largest in the stochastic environment, mothers may have used a conservative bet hedging strategy to produce primarily large offspring of high quality to withstand adverse conditions^22^. Alternatively, the selective environment for mothers in the stochastic environment may have been more similar to actual conditions experienced in the wild for this population e.g. abrupt changes in temperature direction at various time points^29^, thus, results may reflect past selection on reproductive output^6, 43^. Within-female variability of egg size was also higher than among-female variability for females acclimated to 21 °C, which is opposite to that found in a previous study, where constant stress at 21 °C was thought to exacerbate differences among females^29^. The opposing results may reflect the different types of acclimation experienced by females in the two experiments^49^. In the earlier study, females spent their entire lives at 21 °C, and this developmental acclimation to chronic stress may have allowed differences among females to accumulate over time. Here, acclimation was acute (only during the last weeks of reproductive conditioning), and may reflect a more typical, short-term stress response that was similar among females^70^. Taken together, that egg size plasticity occurred with only a few weeks of acute acclimation suggests that stickleback females can fine-tune resource allocation ‘decisions’ just prior to egg laying, even if basal egg size determination is based on accumulated lifetime exposure or possible early-life exposure to different temperatures^29, 60, 71^.

### Stochastic environments promote large offspring body size

Offspring grew best when reared in stochastic environments, regardless of maternal acclimation environment. Overall, offspring were largest in the stochastic environment and smallest when reared at constant 21 °C, whereas offspring reared at 17 °C and the predictably variable environment were similarly sized (Fig. 2). Stochastic environments may promote large size in ectotherms because of the nonlinear and exposure time-dependent relationship between temperature and performance^43^. For instance, chronic exposure to elevated temperature may have detrimental effects on performance, whereas occasional acute exposure to temperatures near or above a critical maximum may benefit organisms by allowing for short bursts of increased activity^6^. For this population, body size and shape variance show nonlinear reaction norms to a temperature range encompassing a 4 °C increase and decrease around the annual mean of 17 °C. Specifically, sibling offspring reared at 13 °C were as large as those reared at 17 °C, but rearing at 21 °C resulted in the common finding of smaller size at elevated temperature^46, 72^. In the current study, sporadic exposure to not only (relatively) extreme high temperatures^73^ but also to low temperatures (14 °C) that favour fast growth in this population may have contributed to overall larger body sizes in the stochastic environment treatment. Likewise, nonlinear or non-additive effects of temperature on performance may also explain why offspring in the predictably variable environment were not intermediate in size between the constant 17 °C and 21 °C environments. Rather, offspring that were exposed to weekly fluctuations between the two temperatures were similarly sized to offspring reared at 17 °C, and both were considerably larger than offspring reared at 21 °C. Short-term exposures to high temperature during development can increase both optimal temperature and maximal growth rate at the optimum via thermal hardening^33^. For example, diurnal fluctuations in temperature have been shown to increase heat shock protein synthesis and heat tolerance, and reduce maximum metabolic rates^43^. Here, the time scale of temperature exposure may have altered the thermal sensitivity of offspring growth performance curves, with week long exposures to elevated temperature being long enough to induce physiological adjustments, but not too long as to lead to performance declines.

Offspring body size was not influenced by an overall effect of maternal acclimation environment or TGP (an interaction between maternal and offspring environments; Table 2). This is in contrast to two previous studies using stickleback females acclimated to constant environments^29, 48^. In the first study, acute acclimation of females did not result in egg size plasticity, so differences in offspring growth were not influenced by initial size. Rather, maternal transgenerational effects on offspring size were mediated by the inheritance of optimised mitochondria^48^. In the second study, developmental acclimation of females led to egg size plasticity, influencing offspring size, but TGP was still present, in that smaller eggs from 21 °C mothers grew to become relatively larger offspring^29^. An underlying physiological mechanism was not investigated in the second study, but it is clear that egg size (e.g. yolk quantity) is only one factor contributing to offspring growth trajectories (see also ref. 36). Egg quality mediated by maternal transfer of somatic factors (hormones, cell structures such as mitochondria) and epigenetic variation including heritable gene expression patterns^74, 75^ also play important roles in shaping offspring phenotypes^21, 76^. More studies are needed to determine the conditions (e.g. environmental cue timing and magnitude) under which different types of transgenerational mechanisms are used to transfer adaptive environmental cues to offspring, and if these are population or genotype specific^74^. Also, studies of the molecular and physiological mechanisms underlying TGP are needed to inform links among epigenetic variation, cellular processes, and resulting phenotypic variation^75, 77, 78^. Here, the period of acute acclimation was two weeks shorter than that used in a previous study^48^, so it may simply be that females did not experience environmental cues long enough to mount a response (see also ref. 79). Nevertheless, the shorter acclimation period was sufficient to induce egg size plasticity. Random variation of selective factors (e.g. SST) in seasonal environments across years^31^ may have contributed to the faster ‘ripening’ of females during the reproductive conditioning phase. Indeed, SSTs were unusually warm during the winter preceding the experiment when adult fish were caught (data available at www.cosyna.de). As a cautionary note, GLMMs that did not include the G × E interaction term as a random effect showed a significant Dam °C × offspring °C interaction. However, this indication for TGP was spurious, as it was driven by smaller mean size in the stochastic environment for offspring of 21 °C mothers (seen in Fig. 2), and not by larger offspring size in the matching maternal environment (indicative of adaptive TGP), highlighting the need to account for effects of G × E into analyses of TGP^19^.

### Within-generation plasticity can override transgenerational plasticity

Genotype by environment interactions may have contributed to the lack of TGP detected here. Offspring environments often have larger effects on the resulting offspring phenotypes than TGP^49, 74^, as strong environmental cues experienced by offspring can override parental effects^28^. Maternal effects are expected to be strongest in early life and diminish as maternal resources are depleted and offspring genes regulating growth and development are switched on refs 13 and 17. Here, maternal effects were present at the egg stage (Fig. 1, Table 1) and some families did show evidence for adaptive TGP at 21 °C in the early growth phase (e.g. reaction norms between 17 °C and 21 °C for families 64 × 164, 20 × 125 and 37 × 140 in the Dam 21 °C acclimation treatment at 30d; Fig. 3), but these effects were dampened by other family reaction norms in the opposite direction (genetic variation for TGP), and to an increasing influence over time of genetic effects on offspring phenotypic variation (Table 3). For instance, release of cryptic genetic variation (see below) promoting G × E interactions may have contributed to offspring environment overriding any potential maternal influence on reaction norms.

Within-generation plasticity (G × E interaction) had the strongest influence on offspring size, and most importantly, indicates that some families perform better in warmer or more variable environments, and thus could be selectively favoured under future climate change^13, 36^. Phenotypic plasticity is an effective means for organisms to respond to changing environments. Most studies have investigated reaction norms over two constant environments, comparing the response of genotypes to changes in the mean value of some variable^80^, but the need to simulate the increased environmental variability predicted under climate change has recently come to light^6^. Rearing individuals under predictable (e.g. diurnal) and stochastic environmental change may more accurately reflect responses to climate change because many organisms encounter daily fluctuations in temperature, and temperature variability is predicted to affect fitness-related traits to a greater extent than changes to mean temperature alone^6, 33, 36^. Here, families responded differently to not only changes in mean temperature, but also to changes in the variability and predictability of temperature fluctuations. Many (but not all) families responded to a mean increase of 4 °C with decreased body size, whereas predictably variable and stochastic environmental fluctuations led to large differences in phenotypic variation between these environments compared to constant conditions (Fig. 3). Interestingly, family reaction norms also changed depending on the type of environmental predictability, in that offspring size in the stochastic environment differed from that seen in the predictably variable environment, which was itself most similar to ambient (17 °C), constant conditions. Environments that fluctuate in a predictable way (e.g. seasonality) are thought to impose more benign impacts on organisms than ‘noisy’ fluctuating environments^41^, and this was likely reflected in the predictably variable environment. Overall, the presence of G × E interactions indicates that, at least for this population, thermal reaction norms for offspring body size have the potential to evolve^81^, not only to changes in mean temperature, but also to increasing temperature variability.

### Evolutionary potential in a directionally vs. stochastically warming ocean

The marine stickleback population studied here showed heritable genetic variation for body size, indicating that adaptive evolution of thermal reaction norms to environmental change is not constrained by the amount of standing genetic variation (see also ref. 48). Comparable results for this population have been shown for other life history (weight, survival) and morphological (shape) traits^46, 47^. Positive genetic correlations across environments also indicate that the evolutionary potential of offspring size in future conditions may be reliably predicted from current levels of standing genetic variation (see also^47^). In this study, genetic variance and total phenotypic variance of body size differed among offspring rearing temperatures (Table 3). At the earliest growth stage (30d), genetic and phenotypic variance were highest when offspring were reared at 21 °C. After this initial phase, however, genetic and phenotypic variances were highest when offspring were reared in the stochastic environment. Stressful, unfavourable and/or novel conditions are known to contribute to changes in variance components and resulting heritability of traits across environments^35^. Mechanisms include changes to environmental variance, non-additive effects including maternal effects, G × E interactions, and release of cryptic genetic variation^81, 82^. Here, changes to total phenotypic variance may have been influenced by many of these mechanisms. First, environmental variance decreased over time within each of the offspring rearing temperatures, contributing to decreasing total phenotypic variance over time. Second, maternal and other non-additive effects may have contributed to changes in phenotypic variance across environments, however, these could not be partitioned out due to the full-sibling design. Nevertheless, the lack of maternal environment effects (Dam °C) or TGP on offspring size in some but not all families suggests that there is substantial variation for genetic as well as non-genetic maternal effects in the studied population. Third, G × E interactions had a strong influence on changes to V_P_ across environments. Genetic and phenotypic variances also differed between the predictably variable and stochastic environments, again suggesting that the type of environmental predictability^41^ in conjunction with the complex relationship between predictability and G × E^31^ can shape the adaptive potential of a population.

Release of cryptic genetic variation when exposed to a stressful or novel thermal environment likely contributed most to the changes in phenotypic variance found^83, 84^. Here, genetic variance was highest at 21 °C in the earliest developmental stage of offspring, and may reflect an initial stress response to a directional increase in mean temperature^70^ (see also refs 46 and 47). At later growth stages, the highest genetic variances were seen under stochastic environmental change (Table 3). Increased V_G_ in the stochastic environment could also reflect a stress response given that populations likely do not regularly experience such large fluctuations within short time periods, and the magnitude of temperature fluctuations has been shown to influence stress levels in salmonids^36^. Alternatively, the stochastic environment more closely mimicked conditions experienced in the wild than constant environments reflecting the mean thermal regime experienced in nature, resulting in a better representation of the actual phenotypic variance available for selection to act on^3, 5^. For instance, an oceanic stickleback population showed a massive increase in additive genetic variance due to release of cryptic genetic variation when reared under low salinity conditions mimicking the colonisation of freshwater habitats^86^. Taken together, the results of the current study show that changing environmental conditions can release otherwise cryptic genetic variation that can consequently alter the evolutionary potential of a population. Given that short-term temperature fluctuations and extreme events like heat waves are predicted to increase in the near future^4^, stickleback populations must (and do) harbour substantial amounts of standing genetic variation in order to adapt quickly to local environmental conditions.

The extent to which the findings from this study translate to other fish species is difficult to gauge, mostly because few non-commercial species have been studied so intensively from both a genetic and non-genetic perspective. Moreover, very few experiments have simultaneously investigated the role of environmental predictability in evolutionary potential. Additive genetic variance is one source of fitness variation available for selection, but non-additive genetic and maternal environmental effects can also substantially alter evolutionary trajectories^87^. Marine fish species with large population sizes, e.g. herring, often have high standing genetic variation and potential to adapt^88^, but the role of non-genetic effects in their adaptive potential has been less studied, in many cases due to logistic constraints or lack of suitability for breeding and rearing in captivity^14^. Stickleback have a high ability to adapt to changing environmental conditions as evidenced by their cosmopolitan distribution across the Northern hemisphere spanning latitudes from the arctic to the Mediterranean, and concomitant past exposure to a broad range of environmental conditions and selection pressures (e.g. temperature, salinity, season length, habitats, predators^89^). Standing genetic variation and the underlying genomic architecture are thought to contribute to recent adaptive radiations and repeated colonisations from marine to freshwater environments^90^. The marine population studied here has also shown strong maternal environment effects as well as temperature-sensitive contributions of maternal variance to offspring phenotypes^29, 48, 75^, indicating that this population employs a number of different genetic and non-genetic mechanisms to cope with rapid environmental change. Likewise, a series of studies of the spiny damselfish (a tropical fish species inhabiting waters where environmental conditions do not vary greatly) also showed that TGP, WGP, genetic and maternal variance all contribute to the adaptive potential of populations under rapid ocean warming^13, 91^, highlighting that generalisations based on environmental history alone can not be made. Furthermore, TGP in response to experimental manipulations of changes to the mean values of temperature^79^, ocean acidification and hypoxia has been found in a number of other fish species, suggesting that adaptive non-genetic effects (e.g. TGP) may be phylogenetically widespread^20^ and a general phenomenon in fishes^92^.

To conclude, the interplay between genetic and non-genetic effects, environmental predictability and evolutionary potential has received little attention to date, but elegant new studies are accumulating. Quantifying the effects of an increase in both the mean and variability of environmental conditions is necessary to predict the ecological and evolutionary responses of populations to future climate change^36^. Here, rearing stickleback offspring in stochastically varying thermal environments produced phenotypic variation not normally seen in constant environment conditions, suggesting that evolutionary potential may be underestimated when the influence of thermal variability is not taken into account. Ideally, future experiments would manipulate changes to environmental variability in conjunction with directional changes to the mean, both within and across generations, as their interaction(s) has been shown to have the largest impacts on organisms^6^.

## Methods

### Environmental predictability

Sea surface temperatures (SST) and predictability of SST that this population of stickleback experience in the wild were previously characterised using data obtained from the Coastal Observing System for Northern and Arctic Seas data web portal (www.cosyna.de). Temperatures off the west coast of Sylt, Germany (54°79′N, 8°27′E), where the studied population originates, range from approximately 13 °C to 20 °C during the reproductive season (May through August), with a mean summer average around 17 °C and an estimated predictability of 6 to 9 days^29^. The hatching time for eggs from this population ranges from 5 to 7 days depending strongly on water temperature. A SST predictability of 6 to 9 days implies that the thermal environment mothers experience at spawning is a good predictor of the conditions hatched fry will experience in very early life^29^. In the current study, four SST regimes were simulated: 1) 17 °C constant, reflecting average summer conditions, 2) 21 °C constant, reflecting a predicted mean increase of 4 °C under climate warming^1^, 3) predictably variable (Var), simulated by changing between 17 °C and 21 °C every 7 days, and 4) stochastically variable (Stoch), simulated by assigning temperatures encompassing the natural range occurring between May and August (between 14 °C and 23 °C) and duration of temperature (between 1 to 7 days) using a random number generator. Water temperature treatments were regulated in each of four header tanks using aquarium heaters, and temperatures were recorded hourly during the experiment using HOBO Pendant® Temperature/Light Data Loggers (Onset Computer Co., Bourne, MA, USA). Average temperatures over the course of the experiment in the four experimental treatments were 17.83 °C (17 °C), 22.09 °C (21 °C), 19.51 °C (Var), and 19.35 °C (Stoch), indicating that the predictably variable and stochastic treatments had similar mean temperatures and differed only in their variability pattern (Supplementary Fig. S4). Cumulative degree-days in the four experimental treatments were 2976.52 (17 °C), 3688.31 (21 °C), 3257.89 (Var), and 3232.34 (Stoch), again reflecting similar overall thermal conditions in the two fluctuating temperature environments.

### Temperature acclimation and fish crosses

All experimental protocols were approved by the German Animal Welfare Standards Agency (Schleswig-Holstein Ministerium für Energiewende, Landwirtschaft, Umwelt und ländliche Räume (Tierschutz), permit no. V312–72241.123–16), and all methods were carried out in accordance with relevant guidelines and regulations. Wild adult marine sticklebacks were caught by trawling off the coast of Sylt, Germany on 11 February 2015 and brought back to the laboratory. As SST was approximately 5 °C at that time, fish were held in groups of approximately 20 in 25 L aquaria at 5 °C for the first few days, and water temperature was gradually raised to 15 °C over the course of four weeks. On 12 March 2015, approximately 50 fish were randomly assigned to each of the four temperature treatments, with 12–13 fish per 25 L aquaria (4 replicate aquaria per treatment). The light regime was set to 10 L:14D, and was adjusted weekly according to ambient photoperiod conditions throughout the experiment. Adult fish were fed daily with chironomid larvae *ad libitum* and experienced between 6 to 8 weeks of temperature treatment acclimation during their reproductive conditioning phase. Starting 20 April 2015, full sibling families were produced by artificial fertilisation within each of the four treatments over the course of two weeks. Crosses were made using established protocols from previous studies^29, 46–49^. Briefly, standard length (±mm) was used as the measure of female size, and eggs were removed by gently squeezing the abdomen until eggs were released (strip spawning). Males were sacrificed in an excess of MS222, the testes were dissected out, crushed in isotonic non-activating medium^93^, and the solution was applied to eggs. Fertilised egg clutches were left for 30 min. before dividing them into four equally sized split-clutches and assigning these to the four temperature treatments. In total, 69 families were produced with n = 21, 19, 16 and 13 families in the 17 °C, 21 °C, Var, and Stoch treatments, respectively. Note: not all adult females in each treatment environment became gravid during the acclimation phase, leading to an uneven number of families produced in the different treatments.

### Offspring traits

Egg size and clutch size were measured using photographs taken under a dissecting microscope and imaging analysis software (LEICA QWIN, Leica Microsystems Imaging Solutions Ltd, Cambridge, UK). Mean egg size in each family was estimated using the diameter (±0.01 mm) of 10 eggs per split-clutch (n = 40 eggs in total). Clutch size was estimated as the total number of eggs per female (four split-clutches combined). Split clutches were each placed individually in1 L glass beakers containing filtered seawater and an air stone. Beakers were held in water baths heated by header tanks set to one of the four experimental temperature treatments. At 14 d post-hatch, the number of hatchlings in each beaker was reduced to ten. Note: split clutches with max. 12 offspring were not reduced to ten. Water was changed in the beakers every week. At 30 d post-hatch, up to 10 offspring per split-clutch were photographed under a dissecting microscope, and imaging analysis software (LEICA QWIN) was used to measure body size (standard length ± 0.01mm). At this point, the up to10 offspring were transferred to a 2 L aquarium connected to a flow-through seawater supply set at one of the four temperature treatments. Standard length was again measured on the 10 offspring per split-family using digital photographs at 60 d and 90 d post-hatch. Throughout the experiment, juvenile fish were fed daily with live *Artemia* sp. nauplii *ad libitum*.

### Data analyses

All analyses were run in the R statistical environment^94^. Generalised linear mixed effect models (GLMMs) were used to quantify the influence of maternal acclimation environment on egg size, clutch size, and the coefficient of variation (CV) of egg size using *lme* within the ‘nlme’ package. Egg size, clutch size and the CV of egg size were modelled as Gaussian response variables with maternal environment (four temperature treatments) as fixed effects, female size and clutch size (or egg size) as covariates, and female identity (individual) as a random effect. The CV of egg size was estimated in two ways: within-female variability (egg size CV for each female within a treatment, and then the mean of this in each treatment) and among-female variability (CV of mean egg size across all females in each treatment; see also ref. 29). Note: only within-female egg size variability was modelled using GLMM, as there was only one value per treatment for among-female variability. *t*- tests were used to determine significant differences between within- and among-female variability in each treatment.

Offspring body size at the three time points (30, 60 and 90 d post-hatch) was analysed in two ways. First, the influence of TGP and G × E interactions on body size across offspring temperatures was analysed. Second, body size variance components within- and genetic correlations between-offspring temperatures were determined. In the first analysis, GLMMs were run using *lmer* within the package ‘lme4’ due to the nature of the random effects, the nested structure of the family and parental acclimation environment term, and the mixed effect interaction terms. Parental temperature (hereafter referred to as Dam °C as both parents experienced the same environment), offspring temperature, egg size and density were modelled as fixed effects. An overall effect of TGP on offspring body size would be indicated by a significant Dam °C × offspring °C interaction. Family (nested within Dam °C) and the family (Dam °C) by offspring environment interaction (G × E) were modelled as random effects. Significance of the G × E interaction term was determined by model testing using likelihood ratio tests between the full and reduced model implemented in *lmer*. Note: a direct test of genetic variation for TGP (Family × Dam °C × Offspring °C) was not possible due to unavoidable constraints of the nested experimental design e.g. different females in each maternal acclimation environment. For graphical display, residual offspring body size (standard length corrected for density) at 30, 60 and 90 d post-hatch are shown as (1) the mean (±SE) of all families per treatment combination, and (2) family-level reaction norms within each maternal environment.

Variance components and genetic correlations were estimated using a character state approach (treating the same trait measured in different environments as separate traits)^95^ using the ‘MCMCglmm’ package^96^. Models were fit as multivariate GLMMs with offspring body size in the four temperature treatments as Gaussian response variables. Only those families that hatched in all four experimental treatments (n = 31) were included in these analyses. Variance components were calculated for the animal term or FS family term for each environment-specific body size (random term: ~us(trait):animal or ~us(trait):FS family, respectively). Since traits in different environments could not be measured on the same individual, the ~idh(trait):units structure of MCMCglmm was used to estimate covariance between environment-specific body sizes^75^. All final models used contained full-sibling family (FS family) as a random effect as opposed to fitting an animal random effect (which takes into account the resemblance among all individuals in the data set irrespective of their level of relatedness^97^) because the study design contained a single generation with large sibling groups, and the additive genetic and maternal variance components could not be partitioned from total genetic variance due to the FS family design (see also ref. 36). Also, analyses using animal as the random effect showed inflated genetic variance components and heritabilities, and very low environmental variance components compared to models fit using FS family, indicating less bias in estimating genetic effects when using FS family. Dam (individual female) was also not modelled as an additional random effect as maternal variance was redundant within the family effect (i.e. no half-sibling families were produced). Dam °C, offspring °C, egg size and density were modelled as fixed effects. Model fits were assessed by their Deviance Information Criteria (DIC) scores including random effects, whereby a ΔDIC greater than 2 represents a significantly better model fit^98^. Weak but informative priors of half the observed variance were used, and the covariance between temperature-specific body sizes was set to zero to account for measurements stemming from separate individuals (see also ref. 48). Markov chains were run for 400 000 iterations, with a burn-in of 100 000, and every 100^th^ value was kept to generate posterior distributions of random and fixed parameters. Genetic correlations (r_G_) were calculated as the covariance between traits (body size in two temperatures) divided by the square root of the product of both traits. Significance was assessed by estimating the proportion of estimates from the posterior distribution that overlapped zero, with a non-significant r_G_ indicative of a G × E interaction^97^.

## Electronic supplementary material

Supplemental information
