# Supplementary material for: The mean and variance of climate change in the oceans: hidden evolutionary potential under stochastic environmental variability in marine sticklebacks
Source: Sci Rep. 2017 Aug 21;7:8889. doi: 10.1038/s41598-017-07140-9 (PMC5567136; doi:10.1038/s41598-017-07140-9)
Supplement: Supplementary file 1 — Supplemental information [file 41598_2017_7140_MOESM1_ESM.pdf]

Supplementary Information

Title: The mean and variance of climate change in the oceans: hidden evolutionary potential under stochastic environmental variability in marine sticklebacks

Author: Lisa N.S. Shama*

Figure S1. Within-female egg size variability for stickleback (*Gasterosteus aculeatus*) mothers acclimated to constant 17 °C, constant 21 °C, predictably variable temperature and stochastic treatments. Histograms of egg size (mm) frequencies are labelled as Dam °C – female identity (e.g. D17 – 122). Note: only n=40 eggs for each of seven females per maternal acclimation treatment are shown (same females as shown in Fig. 3).

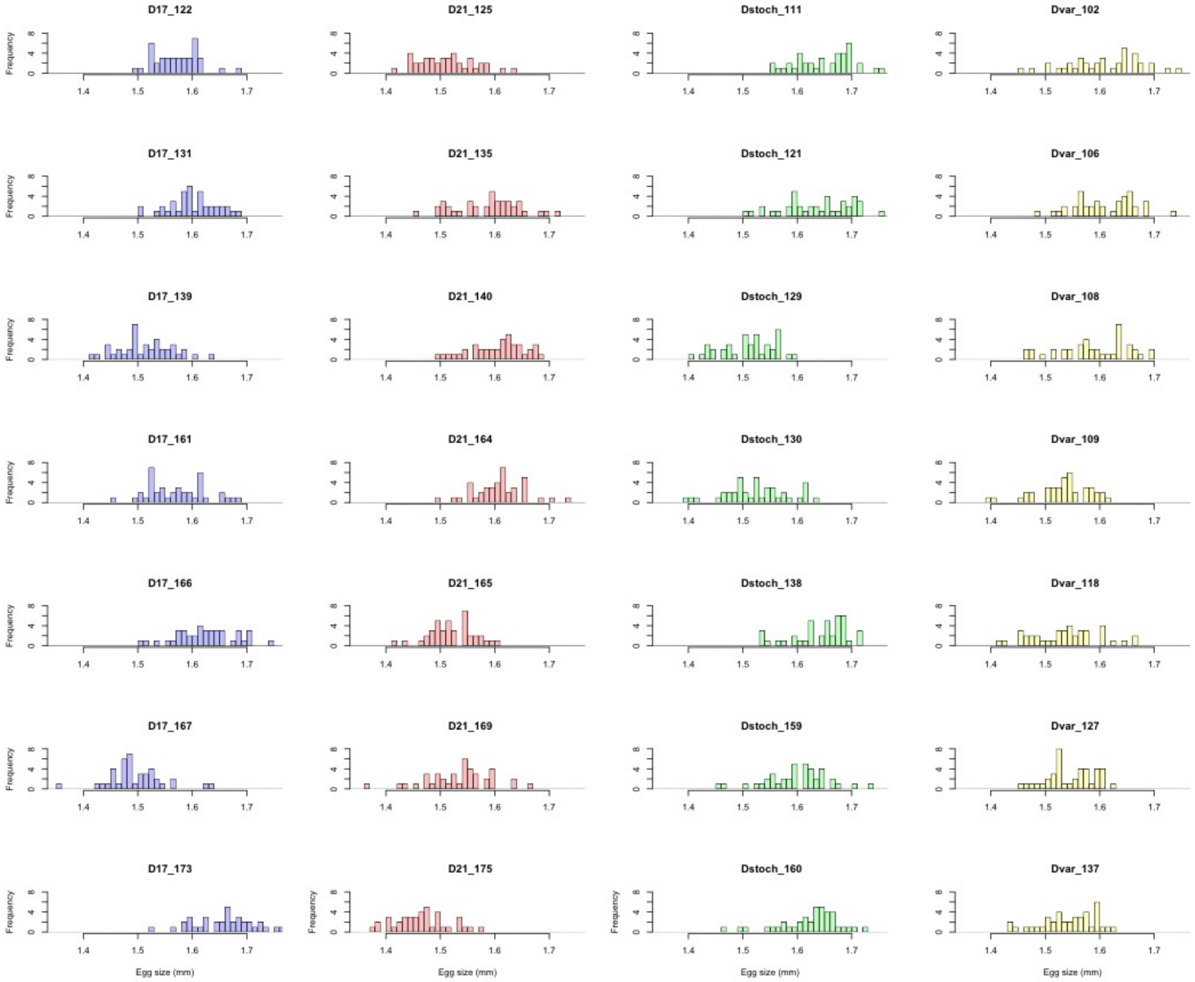

Figure S2. Among-female mean egg size (mm) variability for stickleback (*Gasterosteus aculeatus*) mothers acclimated to constant 17 °C, constant 21 °C, predictably variable and stochastic temperature treatments.

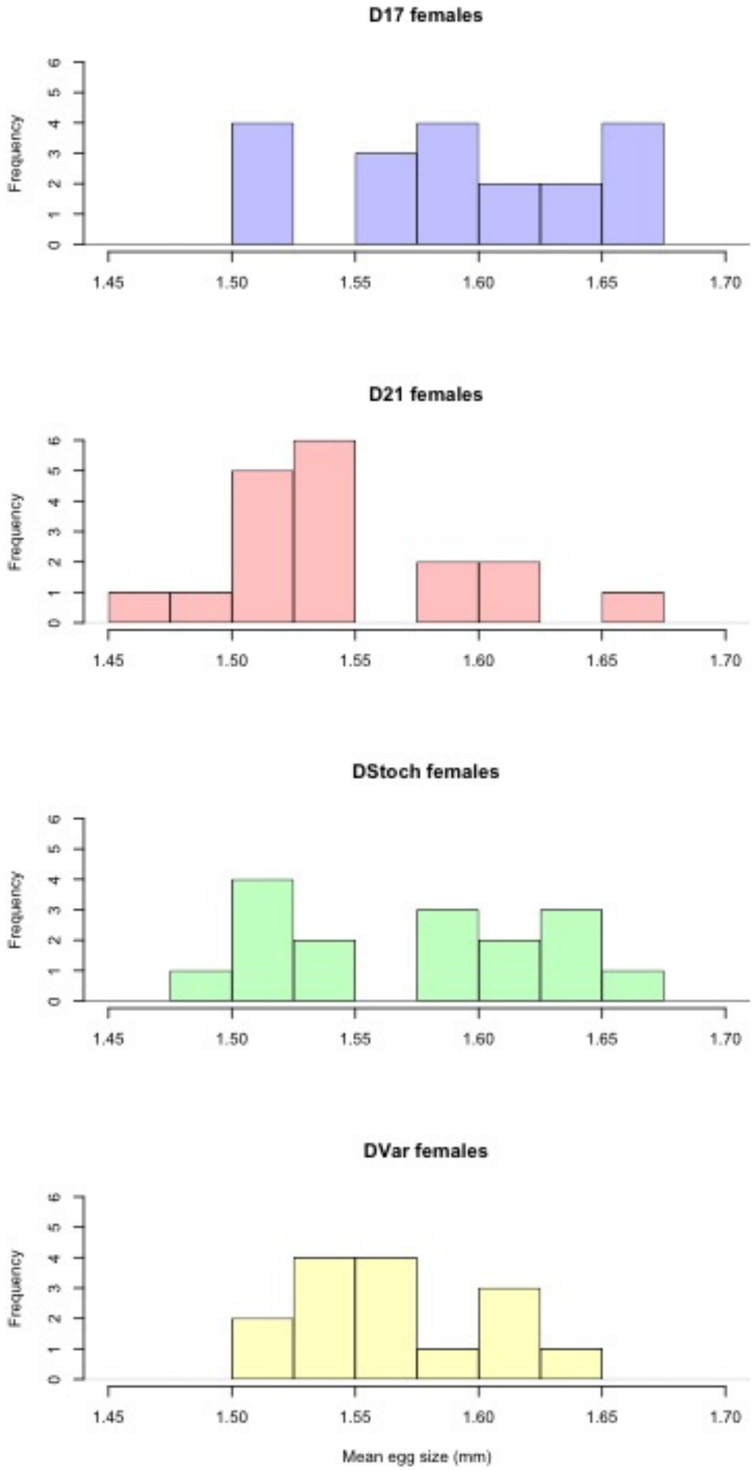

Figure S3. Relationship between density and body size (measured as standard length ± 0.01 mm) for *Gasterosteus aculeatus* offspring reared in the constant 17 °C; open circles, constant 21 °C; closed circles, predictably variable; open triangles, and stochastic; closed squares environments at (a) 30d, (b) 60d, and (c) 90d post-hatch.

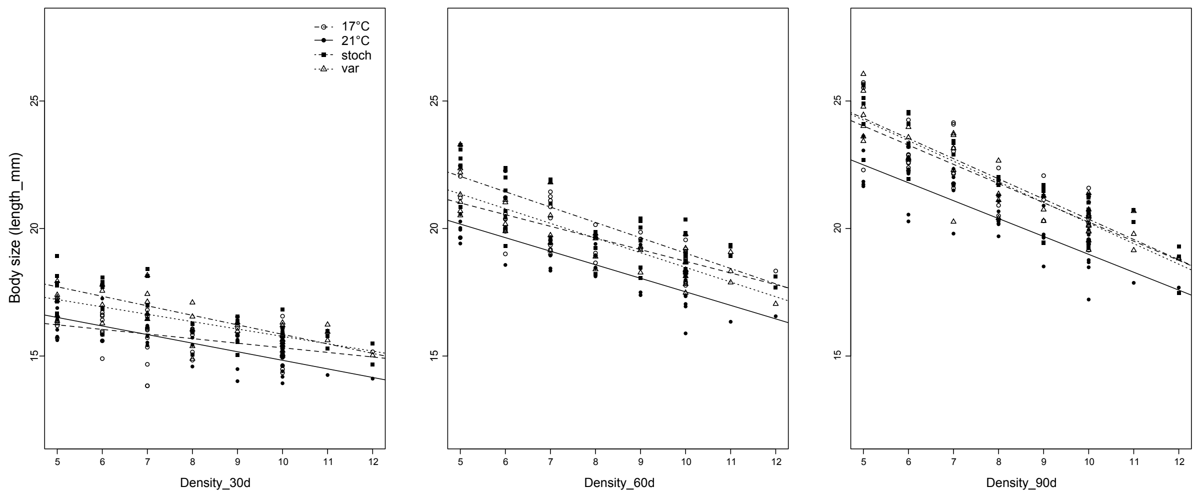

Figure S4. Four experimental temperature treatments (constant 17 °C; blue, constant 21 °C; red, predictably variable; black and stochastically variable; green) used to acclimate mothers during reproductive conditioning and to rear offspring families for 90d post-hatch.

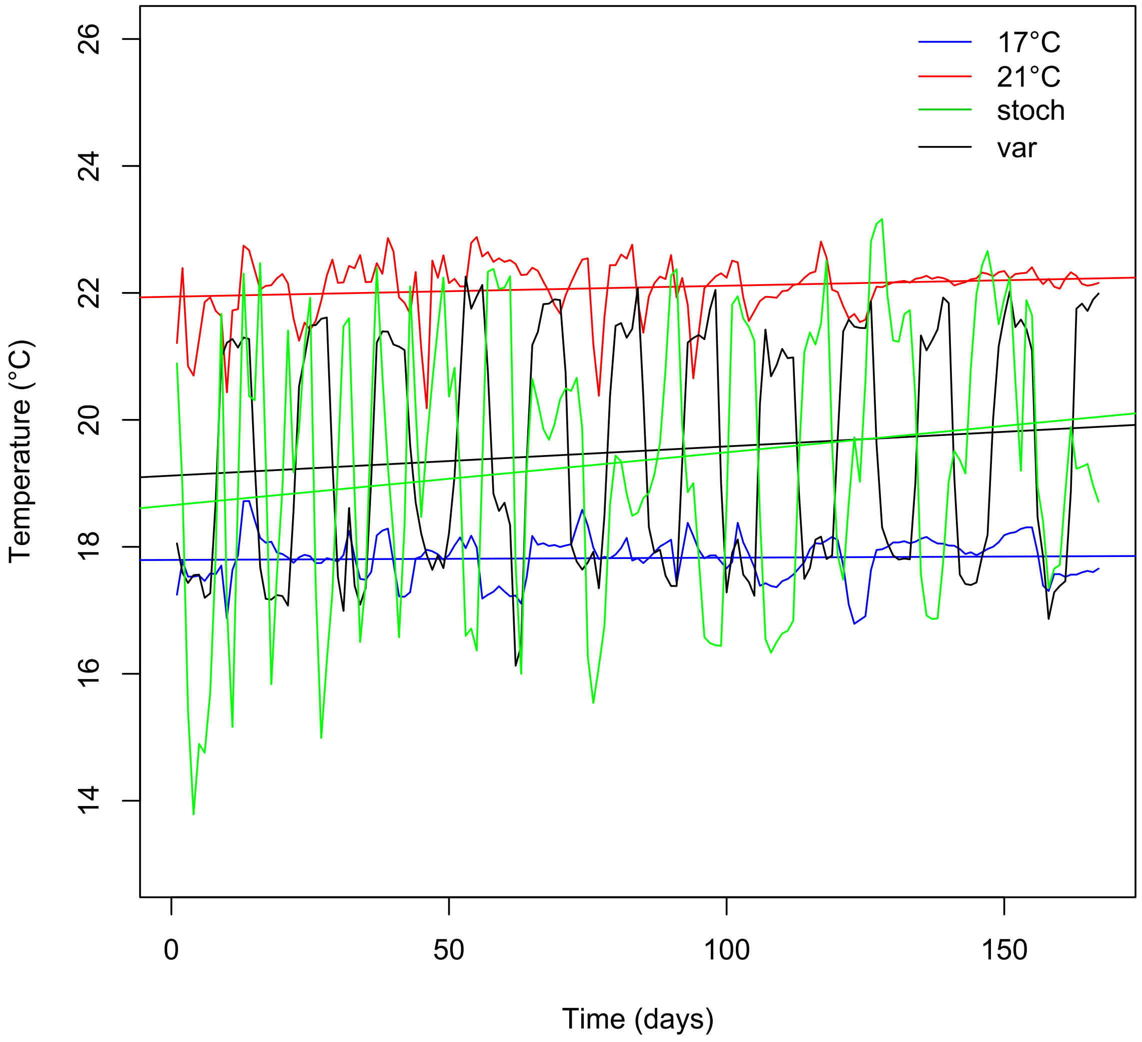

Supplementary Table 1. MCMC GLMM model comparisons and DIC values used to determine the best model fit for stickleback (*Gasterosteus aculeatus*) offspring size at 30, 60, and 90d post-hatch. Random effects are shown in italics and * indicates the best overall model.

Model	Size 30d		Size 60d		Size 90d	
	DIC	ΔDIC	DIC	ΔDIC	DIC	ΔDIC
Family + Density + Egg size + Dam x Offspring °C	2017.56		1648.85		1345.04	
Family + Density + Egg size + Dam °C + Offspring °C	2012.37*	- 5.19	1644.93*	- 3.92	1341.52*	- 3.52
Density + Egg size + Dam °C + Offspring °C	2251.77	+239.4	1850.93	+206.0	1580.52	+238.9
